# Negative refraction and mode trapping of flexural–torsional waves in elastic lattices

**DOI:** 10.1098/rsta.2021.0379

**Published:** 2022-11-28

**Authors:** K. H. Madine, D. J. Colquitt

**Affiliations:** ^1^ Department of Mathematical Sciences, University of Liverpool, Liverpool L69 7ZL, UK; ^2^ Department of Materials Engineering, National Tsing Hua University, Hsinchu City, Taiwan; ^3^ IPHD Programme, National Tsing Hua University, Hsinchu City, Taiwan

**Keywords:** negative refraction, mode trapping, dynamic anisotropy, Green’s functions

## Abstract

We consider the propagation of flexural and torsional waves in a square lattice of Euler–Bernoulli beams. The refraction and reflection of waves across interfaces between two dissimilar lattices is investigated. By carefully controlling the inertial and elastic properties of the lattice elements, we demonstrate that it is possible to induce negative refraction and other associated phenomena. These effects are shown to be broadband and are facilitated by the unprecedented control over wave propagation afforded by the interaction between torsional and flexural waves and the additional freedom associated with the applied forcing. Closed-form analytical findings are accompanied by numerical simulations, which demonstrate negative refraction, unidirectional reflection and mode trapping.

This article is part of the theme issue ‘Wave generation and transmission in multi-scale complex media and structured metamaterials (part 2)’.

## 1. Introduction

Materials capable of negative refraction—in which waves transmitted across interfaces experience a negative angle of refraction—were first postulated by Veselago in the 1960s for electromagnetic media [1]. At that time, limitations in manufacturing resulted in the significance of the work being largely overlooked until the turn of the century, when renewed interest in the design of multi-scale complex media for the control of wave propagation, or metamaterials as they are now known [2], made implementable designs of negative-index materials achievable [3–5].

Historically much of the scholarly literature on negative refraction, and metamaterials more generally, has been devoted to electromagnetic materials, but there is a growing interest in their application to solid mechanics [6]. Although there is scope for the translation of ideas from optical materials to acoustics and elasticity, it must be emphasized that the underlying mathematical and physical framework is very different [7,8]. Metamaterials, despite being a relatively young concept, have already found applications and implementations in many areas, including but certainly not limited to lenses and signal processing for optical media [9–11], sound insulation and wave control for acoustic media [12,13], and seismic protection devices and energy dissipation in elastic media [14–16]. For all of optics, acoustics and elastics, metamaterials have been used for the design and fabrication of cloaking devices [12,17–20], with negative refraction often forming a key component.

There have been numerous contemporaneous studies devoted to controlling the propagation of elastic waves in structured media. Different mechanisms for this control over propagation have been proposed, with much of the control stemming from altering the dispersive properties of the lattice. Examples of this include the use of Dirac cones and parabolic modes as in [21,22], the use of band gaps and band gap defect modes as in [23], and changing the microstructure of the lattice as in [24,25]. The effect of changing the geometry of the lattice has been well studied and is often shown to produce interesting dynamic anisotropy; examples include [26–28]. Elastic lattices and plates have also been combined with additional dynamic elements to create remarkable effects, such as bending waves around corners in [29] and the conversion of reflected modes to waveguide modes [30].

The principle of waveguiding has also formed the basis of elegant elastic cloaking devices, such as in [31], where a novel design of a cloaking device was proposed to guide waves around an inclusion in a flexural plate. This design was then implemented, with the experimental results published in [32] showing significant success at hiding the inclusion. Further cloaking of inclusions on plates and lattices was demonstrated in [33]; notably this work discusses the coupling of torsional and flexural waves, a detail which is often neglected for lattice systems but which forms a cornerstone of the current study. In [33], multiple inclusions are cloaked by stiffening and applying distributed masses to the boundaries of the inclusions. Numerical and experimental results demonstrate that suitably engineered plates with inclusions and subjected to sinusoidal displacements exhibit the same dynamic behaviour as plates without inclusions. The work has significant applications to civil engineering, with particular interest for the field of seismic protection devices.

Discrete interfaces in lattices of thin beams have also been used to induce interesting effects such as negative refraction, filtering and focusing of waves. In particular, the papers [34–36] demonstrated negative refraction across interfaces on lattices which had been divided into regions of beams with differing rotational inertia, modelled as either Euler–Bernoulli or Rayleigh beams. While the Rayleigh model accounts for the rotational inertia of the beam cross-section, these effects are neglected under the Euler–Bernoulli model. The different beam models will, in general, exhibit different dispersive properties on either side of the interface, and this can lead to negative refraction across the interface. It should be noted that in discrete lattices (such as those considered in the present paper), where the ligaments are assumed to be massless and the inertia of the system is concentrated at the junction points, the Rayleigh and Euler–Bernoulli models are equivalent.

In this work, we consider a discrete square lattice of beams and demonstrate that it is possible to induce negative refraction, and other associated phenomena, by controlling the inertial properties of the junction points and elastic properties of the ligaments. In contrast to the earlier works, we emphasize that the present paper considers the rotational inertia of the *beam junctions*, not the internal rotational inertia of the beams. We also account for the coupling of flexural and torsional motions of the lattice. The earlier paper [37] provides a detailed study of the coupling between flexural and torsional interactions for homogeneous infinite two-dimensional lattices of beams. In particular, it was shown that the rotational inertia of the junctions and torsional stiffness of the ligaments provide a refined method for controlling the dispersive properties of the lattice. Moreover, the combination of flexural and torsional interactions provided more freedom in the choice of forcing—not only could classical point forces be imposed, but also point moments, and combinations thereof—which then allowed for the generation of anisotropic, uni-axial and asymmetric waves.

In the present paper, we employ the unique interactions and effects identified in [37] to investigate the refraction and reflection of flexural and torsional waves across interfaces between two dissimilar lattices. Here, we demonstrate that by carefully tuning the elastic and inertial properties of the two lattices, it is possible to generate several interesting phenomena, including negative refraction.

The structure of this article is as follows. In §2 we derive the equations of motion for the system, and in §3 it is shown that the dispersive properties are heavily dependent on the values of the rotational inertia and torsional stiffness, and a variety of different dispersion diagrams are provided that correspond to changing these parameters. In §4, multiple instances of negative refraction are demonstrated, ranging from minor to severe deflection angles. Inclusions in the lattice, formed through altering the dispersive properties of a region, are used as mode trapping devices, and the reflection of waves off the inclusion, in combination with the unique mode shape induced by the forcing vector, can be used to reflect waves and produce waves that propagate only in one direction. It will be shown that the negative refraction and other effects can be induced over broadband frequency and parameter regimes.

## 2. Equations of motion

**Figure 1.**
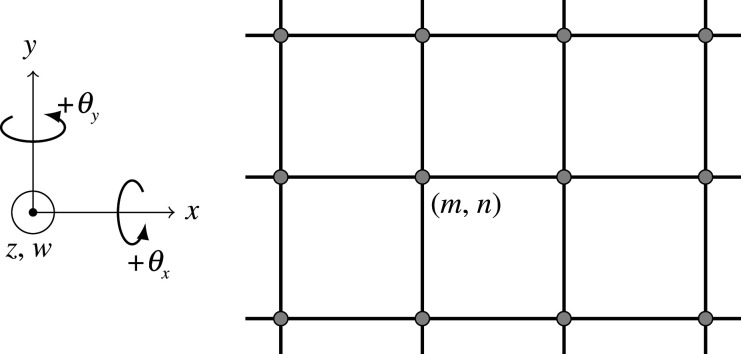
The infinite square lattice with corresponding coordinate axes.

We consider the time-harmonic out-of-plane displacements and rotations of an infinite square lattice, composed of unit-length Euler–Bernoulli beams in the xy-plane as shown in figure 1. The junctions, also referred to as nodes, rotate about the coordinate axes and, as a consequence, torsional and flexural waves become coupled at the junction points. This calls for the introduction of the rotational inertia of the nodes, μ, for rotation induced by the flexural deformations, and the torsional stiffness coefficient of the beams, c, for rotations induced by twisting the beams. The reader is directed to [37] for further explanation of the coupling of flexural and torsional waves along with a detailed derivation of the equations of motion; a brief overview is presented here for convenience.

For the lattice, the junctions are enumerated by $(m,n)\in Z^{2}$ and the displacement amplitude of the nodes is described by the vector $u_{\left(m,n\right)}={\left[w(m,n),\theta_{x}(m,n),\theta_{y}(m,n)\right]}^{\text{T}}\in C^{3}$. The first component of $u_{\left(m,n\right)}$ gives the amplitude of the translational displacement w, while the second and third components give the amplitudes of the rotations experienced by the junctions about the x- and y-axes, respectively. All anti-clockwise angles are taken to be positive in this convention. Firstly, we consider how the junction points are affected by forces and moments induced by the beams lying parallel to the x-axis. For the beams parallel to the x-axis, the flexural deformation is governed by the fourth-order Euler–Bernoulli beam equation

2.1 $$W^{\left(\text{IV}\right)}(x,n,t)+\frac{\rho A}{EJ}\ddot{W}(x,n,t)=0,$$

where overdots denote differentiation with respect to time t and the spatial derivative indicates differentiation with respect to the spatial variable x [38]. For massless beams and time-harmonic displacements of frequency ω, such that $W(x,n,t)=w(x,n)\;e^{\text{i}\omega t}$, the beam equation becomes

2.2 $$w^{\left(\text{IV}\right)}(x,n)=0,$$

where the time-dependence has been suppressed for brevity. We look for solutions w(x,n) in the form of cubic polynomials. The torsional motion generated by twisting in the beams about the x-axis is governed by the second-order equation

2.3 $$\Theta_{x}^{\prime \prime }(x,n,t)-\frac{I_{0}}{JG}{\ddot{\Theta }}_{x}(x,n,t)=0,$$

where the spatial derivative is again taken with respect to x [38]. As with the flexural deformations, the beams are assumed to be massless and we consider time-harmonic displacements. In this case, equation (2.3) simplifies to

2.4 $$\tau_{x}^{\prime \prime }(x,n)=0,$$

where $\Theta_{x}(x,n,t)=\tau_{x}(x,n)\;e^{\text{i}\omega t}$, and we look for solutions $\tau_{x}(x,n)$ in the form of linear polynomials. The coefficients of the polynomials w(x,n) and $\tau_{x}(x,n)$ are determined using boundary conditions [37]. In the normalized form, the equations for the forces and moments induced in the beams are given by

2.5 $$V(x,n)=-w^{\prime \prime \prime }(x,n),\;M(x,n)=w^{\prime \prime }(x,n)\;\text{and}\;T(x,n)=-c\tau_{x}^{\prime }(x,n),$$

where V is the shear force, M is the bending moment arising from flexural deformations and T is the bending moment associated with torsional deformations. All of the above spatial derivatives are performed with respect to the x variable for the beams lying parallel to the x-axis.

The forces and moments arising from beams lying parallel to the y-axis are determined using the same method, solving equivalent equations of motion in terms of the y variable for the polynomials w(m,y) and $\tau_{y}(m,y)$, with the spatial derivatives in that regime performed with respect to the y variable. The forces and moments from the two directions are combined and expressed as stiffness matrices $K_{i}$ acting on the displacement vectors $u_{\left(m,n\right)}$ at the endpoints of the beams. We introduce the forcing vector $f={\left[f_{w},f_{\theta x},f_{\theta y}\right]}^{\text{T}}$, which allows for the application of translational point forces $f_{w}$ out of the plane or point moments $f_{\theta x}$ and $f_{\theta y}$ about the respective axes. The forcing f is applied at the (0,0) node by the use of the Dirac delta function. Combining the external forcing with the induced forces and moments for the x- and y-directions and the inertia of the junctions, we arrive at the equation of motion

2.6 $$[\omega^{2}M+K_{0}]u_{\left(m,n\right)}+K_{1}u_{\left(m+1,n\right)}+K_{2}u_{\left(m,n+1\right)}+K_{3}u_{\left(m-1,n\right)}+K_{4}u_{\left(m,n-1\right)}=f\delta_{\left(0,0\right)},$$

where the diagonal inertia matrix M=diag[1,μ,μ] contains the unit mass and rotational inertia μ of the junctions and the stiffness matrices are detailed in appendix A. Applying the discrete Fourier transform,

2.7 $$u^{F}(k_{1},k_{2})=\sum_{(m,n)\in Z^{2}}\exp(-\text{i}k_{1}m-\text{i}k_{2}n)\;u_{\left(m,n\right)}$$

with spectral parameters $k_{1}$ and $k_{2}$, to equation (2.6) yields the equation of motion in Fourier space,

2.8 $$S(\omega ,k_{1},k_{2})u^{F}(k_{1},k_{2})=f,$$

where

$$S(\omega ,k_{1},k_{2})=[\omega^{2}M+K_{0}+{\text{e}}^{\text{i}k_{1}}K_{1}+{\text{e}}^{\text{i}k_{2}}K_{2}+{\text{e}}^{-\text{i}k_{1}}K_{3}+{\text{e}}^{-\text{i}k_{2}}K_{4}].$$

Multiplying equation (2.8) by $S^{-1}$ and applying the inverse Fourier transform to the $w^{F}(k_{1},k_{2})$ component of the $u^{F}(k_{1},k_{2})$ vector as follows provides the flexural displacement w(m,n) in response to the applied forcing f,

2.9 $$w(m,n)=\frac{1}{4\pi^{2}}\int_{-\pi }^{\pi }\int_{-\pi }^{\pi }w^{F}(k_{1},k_{2})\exp(\text{i}k_{1}m+\text{i}k_{2}n)\;\text{d}k_{1}\;\text{d}k_{2}.$$

Although it cannot be evaluated in closed form, equation (2.9) is readily amenable to standard quadrature techniques and can be evaluated numerically, as was done in [37]. In §4, we will employ finite-element techniques to study uni-axial wave propagation and dynamic anisotropy in combination with interfaces which exhibit negative refraction.

## 3. Dispersive properties

The dispersion equation represents the solvabilty condition of the homogeneous (f=0) equation associated with equation (2.8). In particular, the dispersion equation is

3.1 $$\begin{array}{rl} \det S & \;=144\sin^{2}(k_{1})\zeta (k_{1},k_{2})+144\sin^{2}(k_{2})\zeta (k_{2},k_{1})\\ & \;\;+(24\cos(k_{2})+24\cos(k_{1})+\omega^{2}-48)\zeta (k_{1},k_{2})\zeta (k_{2},k_{1})=0, \end{array}$$

where the repeated function is

3.2 $$\zeta (r,s)=8+2c+4\cos(r)-2c\cos(s)-\mu \omega^{2}.$$

The dispersion equation is cubic in $\omega^{2}$ and therefore has exact solutions. It is important to note that the dispersion equation is dependent on the rotational inertia μ and torsional stiffness c. Changing the values of these parameters can produce very different dispersion surfaces, including significant changes to the width of the finite band gap or, indeed, whether the finite band gap exists at all. In table 1, typical dispersion surfaces for varying values of μ and c have been produced to illustrate the versatility of the lattice.

**Table 1.** Examples of the different dispersion surfaces formed by altering the values of μ and c.

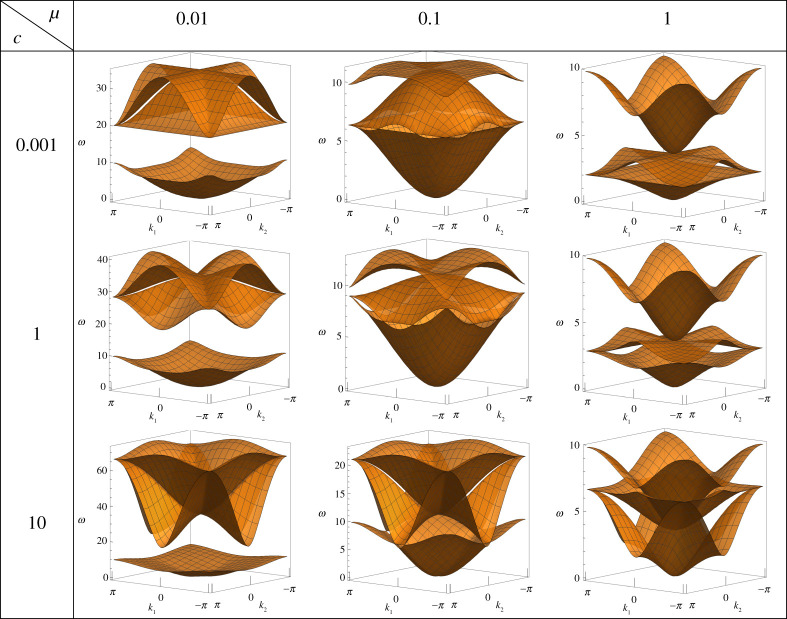

**Figure 2.**
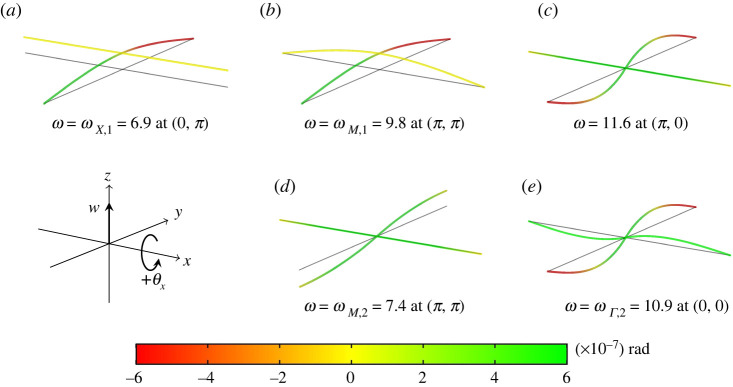
Eigenmodes of the infinite lattice for the values of μ=0.1 and c=1 evaluated at different $\left(k_{1},k_{2}\right)$. The eigenmodes were obtained via a finite-element model of the unit cell, constructed using COMSOL Multiphysics. We emphasize that the colour map represents $\theta_{x}$, the rotation of the beams about the x-axis. (*a*,*b*) Translational eigenmodes; (*c*,*d*) the coupling of flexural and torsional motion; (*e*) flexural motion in two directions that is independent of torsional motion—rather, the entire beam lying along the x-axis rotates without twisting. (Online version in colour.)

As one would expect from a system with three degrees of freedom, the dispersion diagrams have, in general, three dispersion surfaces, each corresponding to a solution of the cubic dispersion equation (3.1). Because of their cumbersome nature, the explicit solutions to the dispersion equation are omitted here in the interest of brevity. We do, however, consider the form of the dispersion equation for three of the high-symmetry points $k=(k_{1},k_{2})$ on the boundary of the irreducible Brillouin zone. In particular, band edges often—but not always—occur at high-symmetry points. In figure 2, we illustrate the eigenmodes of the elementary cell at these points, providing information on the dominant characteristics of the modes (translational, torsional and flexural rotational) for different regimes in the dispersion diagram, or indeed to see if there is any dominant behaviour at all. For a square lattice such as this, the high-symmetry points are Γ=(0,0), X=(0,π) and M=(π,π).

At k=Γ the dispersion equation reduces to

3.3 $$\omega^{2}{\left(12-\mu \omega^{2}\right)}^{2}=0,$$

whence it is immediately apparent that none of the solutions at Γ are dependent on the torsional stiffness of the lattice links. The first, trivial, root $\omega =0≜\omega_{\Gamma ,1}$ indicates that the lattice will never produce a zero-frequency band gap. The second, repeated, root $\omega =2\sqrt{3/\mu }≜\omega_{\Gamma ,2}$ is dependent only on the rotational inertia.

At k=X, the dispersion equation takes the form

3.4 $$(\omega^{2}-48)(4-\mu \omega^{2})(12+4c-\mu \omega^{2})=0$$

and hence has three unique solutions. The first root, $\omega =\sqrt{48}\approx 6.9≜\omega_{X,1}$, is independent of μ and c. The second root, $\omega =2/\sqrt{\mu }≜\omega_{X,2}$, is dependent only on μ, and the third root, $\omega =2\sqrt{(c+3)/\mu }≜\omega_{X,3}$, is dependent on both μ and c. Given that μ and c are positive, these latter two roots maintain their order such that $\omega_{X,3}>\omega_{X,2}$.

Finally, at k=M, the dispersion equation simplifies to

3.5 $$(\omega^{2}-96){\left(4+4c-\mu \omega^{2}\right)}^{2}=0.$$

As one might expect from the previous two cases, there is a first solution $\omega =\sqrt{96}\approx 9.8≜\omega_{M,1}$ with no dependence on either the torsional stiffness or the rotational inertia. The second, repeated, root of the dispersion equation depends on both the torsional stiffness and rotational inertia, $\omega =2\sqrt{(c+1)/\mu }≜\omega_{M,2}$.

From the preceding analysis, the lattice possesses the following set of resonances:

3.6 $$\begin{array}{rl} \Omega & \;=\{\omega_{\Gamma ,1}=0,\;\omega_{\Gamma ,2}=2\sqrt{\frac{3}{\mu }},\;\omega_{X,1}=\sqrt{48},\;\omega_{X,2}=\frac{2}{\sqrt{\mu }},\\ & \;\;\;\;\omega_{X,3}=2\sqrt{\frac{c+3}{\mu }},\;\omega_{M,1}=\sqrt{96},\;\omega_{M,2}=2\sqrt{\frac{c+1}{\mu }}\}. \end{array}$$

We examine the kernel of the matrix S to identify the mode shapes of the lattice at these high-symmetry points. The solutions $\omega_{\Gamma ,1}$, $\omega_{X,1}$ and $\omega_{M,1}$ all have $\text{ ker }S=\{{\left[1,0,0\right]}^{\text{T}}\}$, corresponding to translational modes. Examples of this motion are shown in figure 2*a*, which demonstrates translational plane waves propagating parallel to the y-axis, and figure 2*b*, which demonstrates translational standing waves. We emphasize that the colour map represents changes in the magnitude of $\theta_{x}$, the rotation of the beams about the x-axis, as indicated on the coordinate axes.

The solution $\omega_{X,2}$ corresponds to $\text{ ker }S=\{{\left[0,1,0\right]}^{\text{T}}\}$, which is associated with uniform rotation (without torsion) of one of the lattice links and flexural rotations in the perpendicular link. The solution $\omega_{X,3}$ corresponds to $\text{ ker }S=\{{\left[0,0,1\right]}^{\text{T}}\}$, which is associated with torsional motion in one of the lattice links and flexural rotations in the other, as illustrated in figure 2*c*.

The solutions $\omega_{\Gamma ,2}$ and $\omega_{M,2}$ both have $\text{ ker }S=\{{\left[0,1,0\right]}^{\text{T}},{\left[0,0,1\right]}^{\text{T}}\}$. In this case, the kernel indicates that orthogonal rotational eigenmodes exist at the same frequency. Examples of these modes are provided in figure 2*d*, which shows the coupling of flexural and torsional motion, and figure 2*e*, which demonstrates flexural motion in two directions without torsional deformation in the beam parallel to the x-axis.

Now we shall consider what the above resonances can tell us about the existence, or otherwise, of band gaps. The dispersion equation (3.1) has at most three real non-negative solutions, each one corresponding to a dispersion surface. At Γ and M, the dispersion equation (3.1) has roots of multiplicity two: $\omega_{\Gamma ,2}$ and $\omega_{M,2}$, respectively, for all geometrical and material parameter values. These repeated roots correspond to intersections of dispersion surfaces and, therefore, the lattice exhibits at most one finite band gap. In addition, if there exists a band gap, then the roots $\omega_{\Gamma ,2}$ and $\omega_{M,2}$ cannot belong to the lowest dispersion surface because of their multiplicity. Moreover, since $0=\omega_{\Gamma ,0}<\omega_{\Gamma ,2}$ and $\omega_{\Gamma ,2}$ is a repeated root, if a finite band gap exists then it must be bounded from below by the first dispersion surface, since $\omega_{\Gamma ,0}$ must lie on the lowest dispersion surface.

Inspecting the parameter-dependent resonances of Ω, we see that depending on whether c≶2, either

$$\omega_{X,2}<\omega_{M,2}<\omega_{\Gamma ,2}<\omega_{X,3}\;\text{or}\;\omega_{X,2}<\omega_{\Gamma ,2}<\omega_{M,2}<\omega_{X,3}.$$

If the rotational inertia is chosen to be μ≥1/24, then the root $\omega_{X,2}$ coincides with or is greater than the root $\omega_{M,1}=\sqrt{96}$. Therefore, for μ≥1/24 the upper frequency limit of the high-symmetry points is either $\omega_{M,1}$ or $\omega_{X,3}$ depending on c and the exact choice of μ, and can be an *approximate* lower limit for the semi-infinite band gap. This can be seen by comparing the second and third columns of table 1, and we state ‘approximate’ for good reason. It is important to consider the work of [39], which investigates the relationship between the band edge and the high-symmetry points; in particular, the band edge does not always occur at a high-symmetry point, and indeed in the present work this phenomenon has been observed for a limited selection of μ and c where the upper dispersion surface deviates above the values at the high-symmetry points. In light of this and the sensitivity of the dispersion equation to the material parameters, the entire dispersion diagram must be inspected for each chosen value of μ and c. However, the deviations of the band edge occur so rarely, and are small enough, that the solutions at the high-symmetry points are either the true band edge or a very close approximation.

Considering now situations where μ<1/24, splitting of the solutions $\omega_{M,1}<\omega_{X,2}$ begins to occur and, with this, finite band gaps appear. It is interesting to note that the maximum width of the band gap depends only on μ; in particular, the band gap frequency range is at most

$$\sqrt{96}<\omega <\frac{2}{\sqrt{\mu }},$$

allowing for wider band gaps as μ decreases. Examples of dispersion diagrams with band gaps are presented in the first column of table 1. Again referring to [39] and the sensitivity of the lattice to the parameters, one should always consider the entire dispersion diagram for the chosen parameters.

Regarding the choice of the parameter values for μ and c, we must also take into account their feasibility. The non-dimensional torsional stiffness of the lattice links is

$$c=\frac{GJ_{\tau }}{EJ},$$

where E is Young’s modulus, G is the shear modulus, J is the second moment of inertia and $J_{\tau }$ is the torsion constant [38]. For prismatic beams of cross-sectional area A, J and $J_{\tau }$ are both of order $A^{2}$, and G/E=2(ν+1), where ν is Poisson’s ratio. Therefore, typical values of c range from zero to O(1). The moment of inertia for uniform lattice nodes is proportional to the square of the cross-sectional area; therefore, in the infinitesimal limit as the cross-sectional area vanishes while holding the mass constant, 0<μ≪1.

Returning to the regime of μ<1/24 where a finite band gap exists, the translational eigenmodes from the $\omega_{\Gamma ,1}$, $\omega_{X,1}$ and $\omega_{M,1}$ solutions all appear on the lower dispersion surfaces. Studying the other modes of the lattice in this regime, we conclude that the lower dispersion surfaces below the band gap, such as those in the first column of table 1, are dominated by translational motion. Similarly, the eigenfrequencies in Ω corresponding to flexural and torsional rotations all occur in the upper pass band, so we conclude that rotational motion dominates above the finite band gap.

Considering μ≥1/24, such as in the second and third column of table 1, it is no longer a simple matter to determine which type of motion—translational, flexural rotation or torsional rotation—dominates a given dispersion surface. Taking as an example the dispersion diagram with μ=0.1 and c=1 from table 1, whose eigenmodes are illustrated in figure 2, all surfaces have a mixture of translational and rotational eigenmodes at the high-symmetry points, so we can only conclude that there is no dominant mode for each surface in this case. Furthermore, given the sensitivity of the dispersion diagrams to the rotational inertia and torsional stiffness, the fact that the system is fully coupled, and the size of the parameter space for μ and c, it is not possible to provide a generalization that associates a particular dispersion surface with a dominant type (translational, flexural rotation, torsion) of motion.

Even when the boundaries of the pass bands are invariant, such as all of the lower dispersion surfaces from the first column of table 1, the shape of the surface and its slowness contours still depend on the values of μ and c. Therefore, the parameters μ and c allow for a great deal of control over the direction of propagating waves in the system. The slowness contours are typically very sensitive to changes in the two parameters, so it is common to find slowness contours with completely different shapes for the same value of ω. We will make use of this capability in §4 to demonstrate a number of interesting features, including negative refraction.

## 4. Refraction and reflection at the interface

A finite-element model of the lattice using 201×201 beams was constructed using COMSOL Multiphysics. Absorbing regions adjacent to the boundaries of the computational window were implemented using Rayleigh damping, in order to prevent artificial reflections from the boundary and to simulate the infinite lattice. The lattice was divided into two halves vertically, and an interface was formed by altering the values of the rotational inertia and torsional stiffness on each side of the divide. Continuity of forces, moments, displacements and rotations was imposed across the interface between the two lattices.

### (a) Negative refraction

As discussed in §3, the lattice’s propagating frequencies, dispersion surfaces and therefore slowness contours and principal directions of propagation can be completely altered depending on the choice of the rotational inertia and torsional stiffness. Indeed, the lattice is so adaptable that we are able to choose the shape of the slowness contour we desire through altering the constants and the forcing frequency. The nature of the forcing vector allows the application of moments about the x- and y-axes and translational forces along the z-axis; combined with carefully selected slowness contours, it is straightforward to generate highly localized wave modes along a desired path.

For all figures in this section, the slowness contours for the frequency of the applied forcing have been provided for two halves of the lattice; in all cases the black slowness contour corresponds to the left-hand side (LHS) of the interface and the red slowness contour corresponds to the right-hand side (RHS) of the interface. The parameters $\mu_{1}$ and $c_{1}$ are the rotational inertia and torsional stiffness for the LHS of the interface, with $\mu_{2}$ and $c_{2}$ corresponding to the RHS.

**Figure 3.**
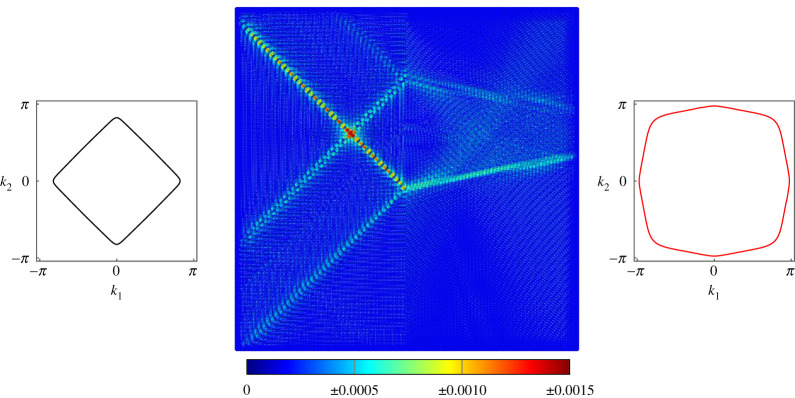
Negative refraction at the interface on a lattice of Euler–Bernoulli beams. The beams on the left have $c_{1}=10$ and $\mu_{1}=1$, while the beams on the right have $c_{2}=0.001$ and $\mu_{2}=0.1$. Forcing of frequency ω=7 is applied using the vector $f={\left[0,-1,-1\right]}^{\text{T}}$. (Online version in colour.)

The first example of negative refraction is given in figure 3. The lattice is subjected to forcing of frequency ω=7 on the LHS of the interface. The forcing vector $f={\left[0,-1,-1\right]}^{\text{T}}$ applies simultaneous point moments about the x- and y-axes. With the diamond-shaped slowness contour, this induces the primary diagonal wave mode propagating along the lines −π/4 and 3π/4. The wave is partially reflected at the boundary and the transmitted wave experiences strong negative refraction.

**Figure 4.**
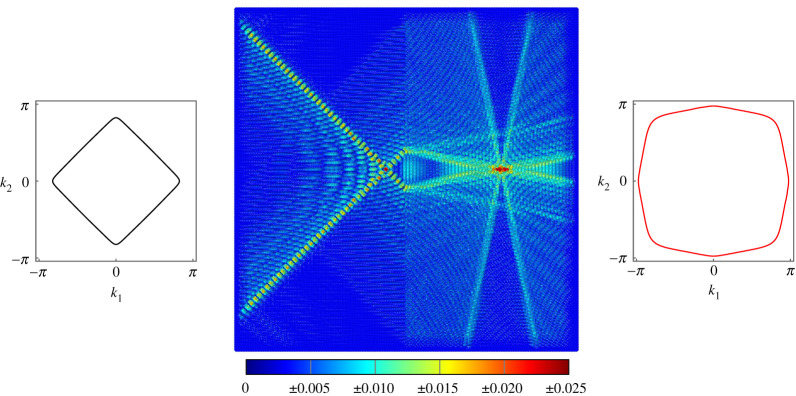
Negative refraction at the interface on a lattice of Euler–Bernoulli beams. The beams on the left have $c_{1}=10$ and $\mu_{1}=1$, while the beams on the right have $c_{2}=0.001$ and $\mu_{2}=0.1$. Forcing of frequency ω=7 is applied using the vector $f={\left[0,0,-1\right]}^{\text{T}}$. (Online version in colour.)

For the same values of μ, c and forcing frequency, another example of negative refraction is generated in figure 4 by shifting the forcing point to the RHS of the interface and changing the forcing vector to $f={\left[0,0,-1\right]}^{\text{T}}$, which applies a point moment about the y-axis. The negative refraction observed in this case is so strong that the magnitude of the angle of refraction is greater than the angle of incidence, and the two transmitted modes cross and constructively interfere, giving the appearance of a false secondary forcing point.

**Figure 5.**
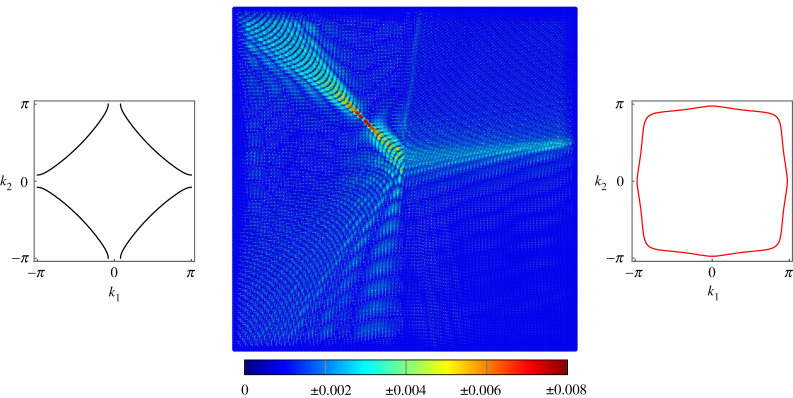
Negative refraction at the interface on a lattice of Euler–Bernoulli beams. The beams on the left have $c_{1}=0.1$ and $\mu_{1}=1$, while the beams on the right have $c_{2}=0.1$ and $\mu_{2}=0.1$. Forcing of frequency ω=7 is applied using the vector $f={\left[0,-1,-1\right]}^{\text{T}}$. (Online version in colour.)

In figure 5, we produce similar negative refraction to that seen in figure 3. We use the same forcing vector and forcing frequency ω=7 but different values of the rotational inertia and torsional stiffness on the two sides of the interface. This demonstrates not only the versatility of the lattice but also that negative refraction is seen for a wide array of parameter combinations.

**Figure 6.**
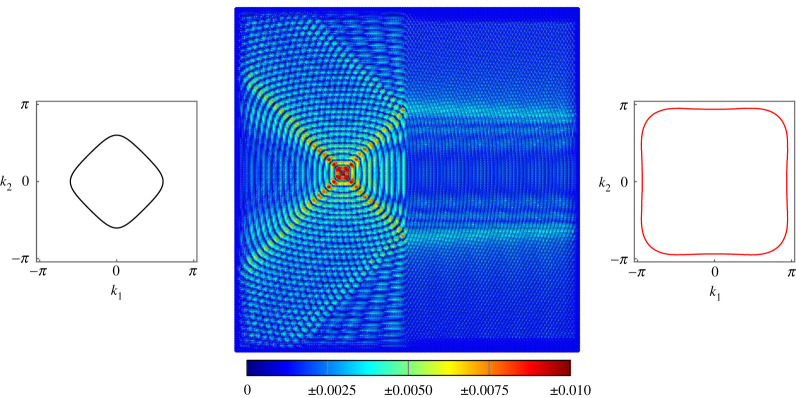
Negative refraction at the interface on a lattice of Euler–Bernoulli beams. The beams on the left have $c_{1}=0.1$ and $\mu_{1}=1$, while the beams on the right have $c_{2}=0.1$ and $\mu_{2}=0.1$. Forcing of frequency ω=6 is applied using the vector $f={\left[-1,0,0\right]}^{\text{T}}$. (Online version in colour.)

Another example of negative refraction is given in figure 6, using the same values of μ and c on both sides of the interface as figure 5 but for the forcing frequency ω=6 and forcing vector $f={\left[-1,0,0\right]}^{\text{T}}$. This demonstrates that the negative refraction is a broadband effect and is not limited to a narrow frequency range.

**Figure 7.**
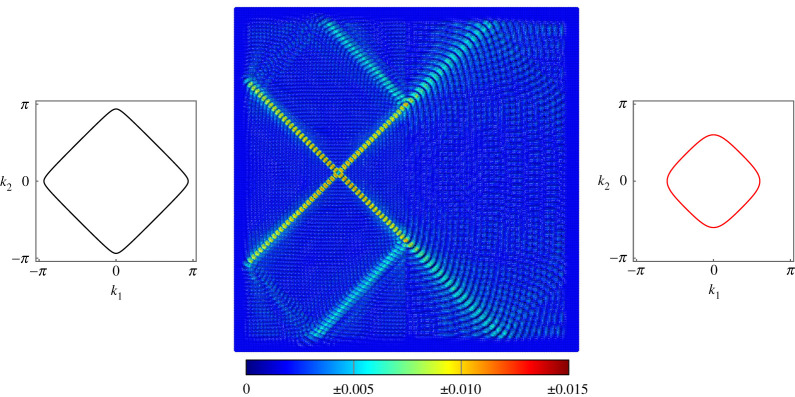
Transmission and reflection at the interface on a lattice of Euler–Bernoulli beams. The beams on the left have $c_{1}=10$ and $\mu_{1}=0.1$, while the beams on the right have $c_{2}=0.1$ and $\mu_{2}=1$. Forcing of frequency ω=6 is applied using the vector $f={\left[-1,0,0\right]}^{\text{T}}$. (Online version in colour.)

Finally, we show that for certain parameter combinations the lattice will produce transmitted waves with no refraction. In figure 7, the similar slowness contours for the two sides of the interface allow the wave to be transmitted across the boundary, with significant reflection but without refraction. This demonstrates that generating the negative refraction still requires careful tuning of the lattice parameters and choice of the slowness contours.

### (b) Beam splitting

**Figure 8.**
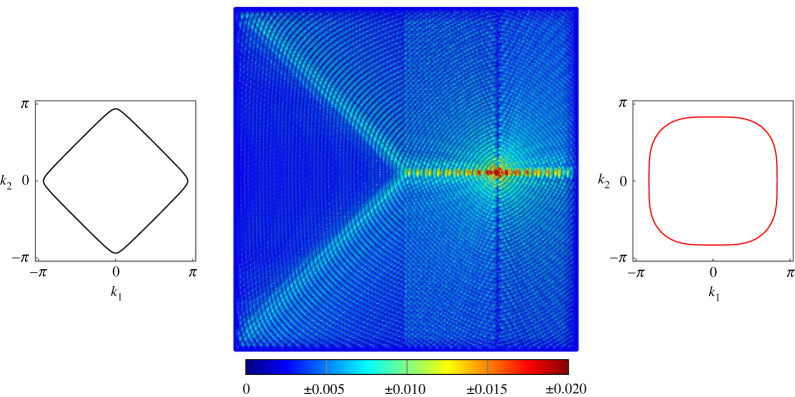
Beam splitting at the interface on a lattice of Euler–Bernoulli beams. The beams on the left have $c_{1}=10$ and $\mu_{1}=0.1$, while the beams on the right have $c_{2}=0.1$ and $\mu_{2}=0.01$. Forcing of frequency ω=6 is applied using the vector $f={\left[0,0,-1\right]}^{\text{T}}$. (Online version in colour.)

The interface can also be used to divide the transmitted wave, which we refer to as ‘beam splitting’. This phenomenon can be produced by choosing μ and c such that the slowness contours for the two sides of the interface have completely opposing preferential directions, inducing a wave mode that is normal to the interface, as was done in figure 8. In this instance, the forcing vector $f={\left[0,0,-1\right]}^{\text{T}}$ is used on the RHS to induce a uni-axial wave mode that propagates in both directions along the x-axis. When the wave reaches the interface, it becomes split and propagates along the lines 3π/4 and −3π/4, as shown to be the preferred directions of travel from the diamond-shaped slowness contour on the LHS.

### (c) Inclusions and mode trapping

**Figure 9.**
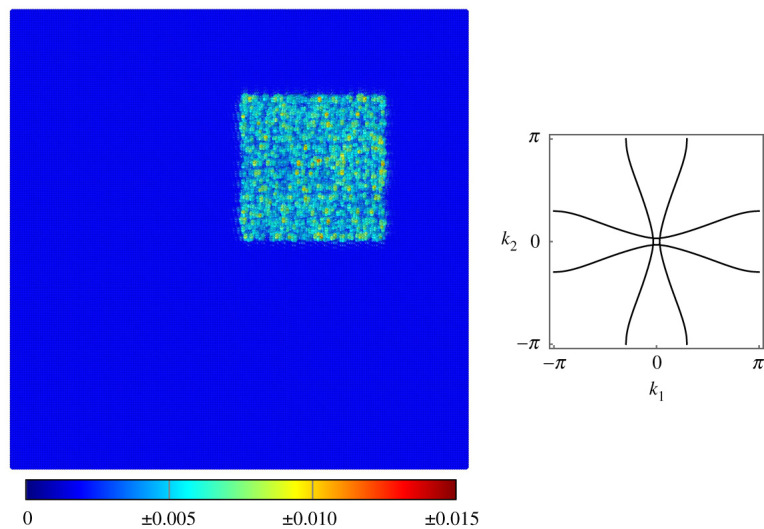
Mode trapping inside an inclusion on a lattice of Euler–Bernoulli beams. Forcing of frequency ω=11 is applied inside the inclusion using the vector $f={\left[-1,0,0\right]}^{\text{T}}$. The beams of the surrounding lattice have $c_{s}=0.1$ and $\mu_{s}=1$, which produces a band gap for this frequency, while the beams in the inclusion have $c_{\text{inc}}=0.1$ and $\mu_{\text{inc}}=0.1$, producing a propagating frequency. Inset shows the slowness contour for the inclusion. (Online version in colour.)

An inclusion in the lattice can be used as a method of mode trapping in order to isolate mechanical vibrations. We use $\mu_{\text{inc}}$ and $c_{\text{inc}}$ to refer to the rotational inertia and torsional stiffness of the inclusion and use $\mu_{s}$ and $c_{s}$ to refer to the parameters for the surrounding lattice. As was shown in §3, the lattice often has a finite band gap for values of μ<0.1. The presence of the band gap also in general forces the upper pass band into much higher-frequency regimes. Therefore, it is straightforward to find values of $\mu_{s}$ and $c_{s}$ which produce a band gap for a chosen ω, while choosing $\mu_{\text{inc}}$ and $c_{\text{inc}}$ such that ω is a propagating frequency. An example of this mode trapping is provided in figure 9; the mode experiences multiple reflections from the boundary of the inclusion but does not propagate into the surrounding lattice.

**Figure 10.**
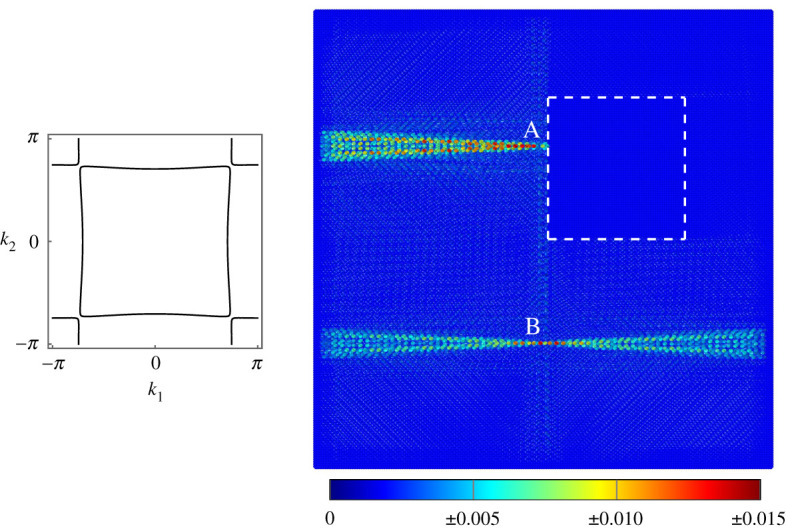
At point A, an inclusion is used as a boundary to reflect uni-axial waves on a lattice of Euler–Bernoulli beams, producing unidirectional reflection. The same forcing is applied at point B away from the inclusion to produce a uni-axial wave. The forcing is applied using the vector $f={\left[0,0,-1\right]}^{\text{T}}$ and frequency ω=24. The beams of the surrounding lattice have $c_{s}=0.1$ and $\mu_{s}=0.01$, which produces a propagating wave, while the beams in the inclusion have $c_{\text{inc}}=0.1$ and $\mu_{\text{inc}}=1$, which produces a band gap for this frequency. Inset shows the slowness contour for the surrounding lattice. (Online version in colour.)

The inclusion can also be used as a method of forming highly localized waves that propagate in one direction. As in figure 8, by choosing $\mu_{s}$, $c_{s}$ and ω to produce a slowness contour that is approximately square and using the forcing vector $f={\left[0,0,-1\right]}^{\text{T}}$, we can generate a uni-axial wave mode that propagates along the positive and negative x-directions. We then choose the combination of $\mu_{\text{inc}}$ and $c_{\text{inc}}$ such that ω lies in the band gap for the inclusion. The inclusion is used as a solid boundary from which to reflect the uni-axial wave, thus producing a wave that propagates in one direction. This method is demonstrated in figure 10 for the forcing point labelled A, which is reflected off the inclusion, with the resulting wave travelling only to the left. For comparison, the same forcing is applied at point B away from the inclusion without reflection and so becomes the familiar uni-axial wave.

In the same manner, the forcing vector $f={\left[0,-1,0\right]}^{\text{T}}$ could be used to generate a wave mode that propagates in the positive and negative y-directions. Reflecting this mode off an inclusion would produce a unidirectional reflected wave along the y-axis. There are infinitely many choices of $\mu_{s}$, $c_{s}$, $\mu_{\text{inc}}$, $c_{\text{inc}}$ and ω that give dispersive waves inside the inclusion and almost-square slowness contours for the surrounding lattice. Therefore, this effect is not at all limited to a narrow bandwidth of frequencies and is resistant to small deviations in the material parameters inside and outside the inclusion.

## 5. Concluding remarks

In this paper, it is shown that an interface between two lattices with contrasting rotational inertia and torsional stiffness can be used as a means to produce negative refraction, focusing and beam splitting effects. Out-of-plane displacements are studied, and the coupling of flexural and torsional waves is properly accounted for at the beam junctions. Other works in which negative refraction has been observed at interfaces on lattices use different constitutive equations for the beams on either side of the interface. This work differs in that the same constitutive equations are used over the whole lattice, and it is the rotational inertia of the junction points and torsional stiffness of the beams which are used as parameters to control the dispersive properties for each side of the interface.

In §2 the equations of motion were derived, and in §3 a range of dispersion diagrams were provided for different values of the rotational inertia and torsional stiffness. It was shown that changing the values of the rotational inertia and torsional stiffness can completely change the shape of the dispersion surfaces and, in turn, produce slowness contours with completely opposing preferential directions, even for the same values of the frequency. The eigenmodes of the lattice at the high-symmetry points of the Brillouin zone were used to demonstrate the coupling of flexural and torsional motion and to demonstrate how the range of propagating frequencies and the existence of the finite band gap are dependent on the chosen values of rotational inertia and torsional stiffness. In §4, we use the rotational inertia and torsional stiffness as parameters to tailor the slowness contours to the shape we desire for each side of an interface on a square lattice of beams. Negative refraction was demonstrated across the interface for different parameter values, forcing frequencies and combinations of applied forcing in the form of point translational forces and point moments. An inclusion formed by altering the dispersive properties for a small selection of beams was used to demonstrate mode trapping and unidirectional reflection by reflecting uni-axial waves off the boundary. The negative refraction and general control over the lattice’s propagating waves demonstrated in this work have applications in many areas of physics and engineering, including cloaking, energy harvesting, filtering and seismic protection.

## Data Availability

This article has no additional data.

## Appendix A. The stiffness matrices

The stiffness matrices from equation (2.6) describe the forces and moments applied at the (m,n)th node from the four connecting beams. Each beam contributes two matrices corresponding to the near and far ends of the beam. The use of the Fourier transform in formulating equation (2.8) is equivalent to applying Bloch–Floquet periodicity conditions, and thus the matrices describe the nearest-neighbour interactions over the Brillouin zone in reciprocal space.

The $K_{0}$ matrix is a combination of the four matrices from the near ends of the four beams that connect at $u_{\left(m,n\right)}$ and so is expressed as

A 1 $$K_{0}=\left[\begin{array}{ccc} -12 & 0 & -6\\ 0 & -c & 0\\ -6 & 0 & -4 \end{array}\right]+\left[\begin{array}{ccc} -12 & 0 & 6\\ 0 & -c & 0\\ 6 & 0 & -4 \end{array}\right]+\left[\begin{array}{ccc} -12 & 6 & 0\\ 6 & -4 & 0\\ 0 & 0 & -c \end{array}\right]+\left[\begin{array}{ccc} -12 & -6 & 0\\ -6 & -4 & 0\\ 0 & 0 & -c \end{array}\right].$$

The $K_{1}$ and $K_{2}$ matrices describe the forces and moments from the far ends of the beams lying in the positive x- and y-directions, respectively, and are expressed as

A 2 $$K_{1}=\left[\begin{array}{ccc} 12 & 0 & -6\\ 0 & c & 0\\ 6 & 0 & -2 \end{array}\right]\;\mathrm{and}\;K_{2}=\left[\begin{array}{ccc} 12 & 6 & 0\\ -6 & -2 & 0\\ 0 & 0 & c \end{array}\right].$$

The $K_{3}$ and $K_{4}$ matrices describe the forces and moments from the far ends of the beams lying in the negative x- and y-directions, respectively, and are given by

A 3 $$K_{3}=\left[\begin{array}{ccc} 12 & 0 & 6\\ 0 & c & 0\\ -6 & 0 & -2 \end{array}\right]\;\mathrm{and}\;K_{4}=\left[\begin{array}{ccc} 12 & -6 & 0\\ 6 & -2 & 0\\ 0 & 0 & c \end{array}\right].$$
